# Targeting a systolic blood pressure of <130 mmHg is beneficial in adults with hypertension aged ≥75 years: a systematic review and meta-analysis

**DOI:** 10.1038/s41440-025-02302-z

**Published:** 2025-08-12

**Authors:** Yoichi Nozato, Yume Nohara-Shitama, Takuro Kubozono, Hiroshi Akasaka, Yoichi Takami, Hisatomi Arima, Atsushi Sakima, Koichi Yamamoto

**Affiliations:** 1 Department of Geriatric and General Medicine, The University of Osaka, Graduate School of Medicine, Osaka, Japan; 2 Toyonaka Municipal Hospital, Department of Geriatric and General Medicine, Osaka, Japan; 3 Division of Cardiovascular Medicine, Department of Internal Medicine, Kurume University School of Medicine, Fukuoka, Japan; 4 Department of Cardiovascular Medicine and Hypertension, Graduate School of Medical and Dental Sciences, Kagoshima University, Kagoshima, Japan; 5 Department of Hygiene and Preventive Medicine, Iwate Medical University School of Medicine, Iwate, Japan; 6 Department of Preventive Medicine and Public Health, Faculty of Medicine, Fukuoka University, Fukuoka, Japan; 7 Health Administration Center, University of the Ryukyus, Okinawa, Japan

**Keywords:** Implemental hypertension, Meta-analysis, Older people, Randomized controlled trial, Systematic review

## Abstract

Recent clinical trials have raised important questions regarding optimal blood pressure (BP) targets in older adults with hypertension. In the 2019 Japanese Society of Hypertension guidelines, a systolic BP (SBP) target of <140 mmHg is recommended for individuals aged ≥75 years. However, subsequent randomized controlled trials (RCTs) have shown potential cardiovascular and mortality benefits associated with strict BP targets. We conducted an updated systematic review and meta-analysis to evaluate the efficacy and safety of intensive SBP control (<130 mmHg) compared with less intensive control (≥130 mmHg) in patients with hypertension aged ≥75 years. We searched MEDLINE, Cochrane Library, and Ichushi Web for publications up to May 30, 2024, supplemented by manual searches. Seven RCTs that met predefined eligibility criteria were included in the final meta-analysis. Among patients aged ≥75 years, intensive SBP lowering was associated with significantly reduced risks of composite cardiovascular events (risk ratio [RR]: 0.61, 95% confidence interval [CI]: 0.40–0.94, *p* = 0.03), all-cause mortality (RR: 0.72, 95% CI: 0.56–0.93, *p* = 0.01), and cardiovascular mortality (RR: 0.55, 95% CI: 0.35–0.88, *p* = 0.01), with no increase in serious adverse events (RR: 1.00, 95% CI: 0.93–1.08, *p* = 0.97). Stroke incidence did not differ significantly between groups. Similar results were observed when the analysis was expanded to include studies that enrolled participants aged ≥70 years. These findings support the safety and clinical benefits of targeting an SBP of <130 mmHg in older adults with hypertension.

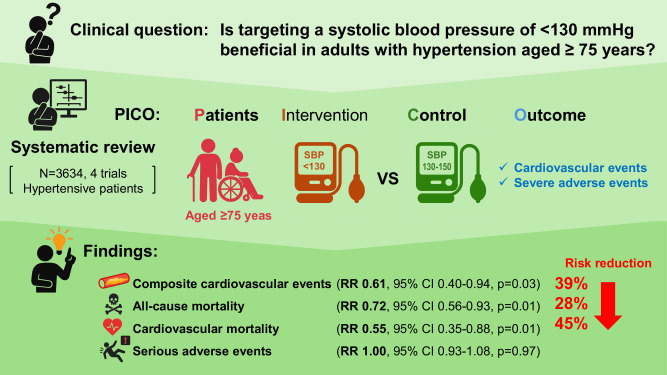

## Introduction

Blood pressure (BP) increases steadily with age, and is driven by progressive arterial stiffening, impaired vasoregulatory mechanisms, and increased sympathetic activity. As a result, hypertension is highly prevalent among older adults, particularly those aged ≥75 years [1, 2]. In Japan, where a super-aged society is a defining demographic feature, optimizing hypertension management in this age group is of critical importance for preventing cardiovascular morbidity and mortality [3, 4].

Traditionally, clinical guidelines have proposed different BP targets for younger and older individuals. The 2019 Japanese Society of Hypertension (JSH) guidelines recommended a systolic BP (SBP) target of <130 mmHg for patients aged <75 years and <140 mmHg for those aged ≥75 years [3]. This is partly due to concerns about treatment tolerability in frail older individuals and the limited number of randomized controlled trials (RCTs) specifically targeting this age group. However, these age-based distinctions in BP targets have sometimes led to confusion in real-world practice, making therapeutic goals less transparent and potentially contributing to under-treatment. In older patients, hypertension management presents a clinical challenge because of the delicate balance between therapeutic benefits and potential harm. Although BP reduction reduces cardiovascular risk, intensive treatment may lead to adverse outcomes, such as acute kidney injury, orthostatic hypotension, falls, or cognitive decline, particularly in frail individuals [5]. Furthermore, evidence regarding which subgroups of older adults truly benefit from strict BP control remains limited, leaving clinicians uncertain about optimal targets. Simultaneously, the older adult population has become increasingly heterogeneous. Many older adults maintain robust physical and cognitive functions in their 80 s and 90 s, reflecting a demographic shift toward healthier aging [6]. This evolution challenges the conventional view that conservative BP targets are universally appropriate in older adults. This highlights the need to re-evaluate hypertension treatment strategies and tailor BP targets based on functional status, comorbidities, and individual risk profiles. In this context, summarizing and integrating the latest evidence on BP goals in older patients with hypertension has become critical for guiding clinical decision-making. The Systolic Blood Pressure Intervention Trial (SPRINT) significantly influenced the 2019 guidelines by showing that intensive BP control reduces cardiovascular events and mortality [7]. However, the use of unattended automated office BP and exclusion of patients with diabetes or prior stroke limit its generalizability in a broader population.

A previous systematic review [8] conducted for the JSH 2019 guidelines demonstrated the clinical benefit of antihypertensive treatments with a target SBP of <140 mmHg in patients aged ≥75 years. Notably, only two of the six RCTs (SPS3 subanalysis [9] and SPRINT subanalysis [10]) had a target BP of <130 mmHg in the intensive group. The remaining three trials (JATOS [11], VALISH [12], and Wei et al. [13]) had a target of <140 mmHg in the intensive group, and one trial (ADVANCE [14]) did not specify an explicit target. In four of these studies, the average achieved BP was >135 mmHg. Subsequently, several randomized controlled trials (RESPECT [15], INFINITY [16]) have reported the clinical benefits of more intensive BP lowering with a target SBP of <130 mmHg even in patients aged ≥75 years, prompting us to conduct a new systematic review. Our primary aim was to assess whether targeting an SBP of <130 mmHg provides superior cardiovascular and mortality benefits compared with less intensive BP targets (≥130 mmHg) in this specific population without increasing the risk of serious adverse events. By focusing on RCTs exclusively involving very old patients and adopted more intensive BP targets, this review provides robust and directly applicable evidence to inform future clinical decision-making in the management of hypertension in older patients.

## Methods

This systematic review and meta-analysis was designed to update a previous meta-analysis [8]. This study was conducted in accordance with the Preferred Reporting Items for Systematic Reviews and Meta-Analyses (PRISMA) guidelines [17]. The review protocol was prospectively developed and registered in PROSPERO (ID: CRD42024552415).

### Eligibility criteria

We included RCTs that compared intensive BP lowering strategies (target SBP, <130 mmHg) with less intensive BP control (target SBP, ≥130 mmHg) in patients with hypertension aged ≥75 years. In line with the previous systematic review [8], which included two RCTs enrolling participants aged ≥70 years with a mean age of ≥75 years (mean age: VALISH trial [12], 76.1 years; trial by Wei et al. [13], 76.5 years), we also incorporated RCTs that targeted participants aged ≥70 years. Eligible studies were required to report at least one of the following outcomes: composite cardiovascular events (i.e., myocardial infarction, stroke, heart failure, and cardiovascular death), all-cause mortality, cardiovascular death, stroke, or severe adverse events, including frailty-related events (falls, fractures, hypotension, and cognitive decline, if available). Primary analysis was performed among the extracted trials that enrolled participants aged ≥75 years. Subsequent analysis was performed among participants aged ≥70 years. Studies involving patients undergoing maintenance dialysis or hypertensive emergencies, including those in the acute phase of stroke, were excluded. Follow-up observational studies of original RCTs were excluded from the meta-analysis.

### Information sources and search strategy

A comprehensive literature search was conducted using the Ovid MEDLINE, Cochrane Central Register of Controlled Trials, and Ichushi-Web between January 1, 2017 and May 30, 2024. Medical Subject Headings and free-text terms were used. Complete search strategies are presented in Supplementary Table 1. We manually searched the reference lists of eligible articles and prior systematic reviews to identify additional studies, including four key trials (the PAST-BP [18], PODCAST [19], RESPECT [15], and ESPRIT [20]).

### Study selection

**Fig. 1:**
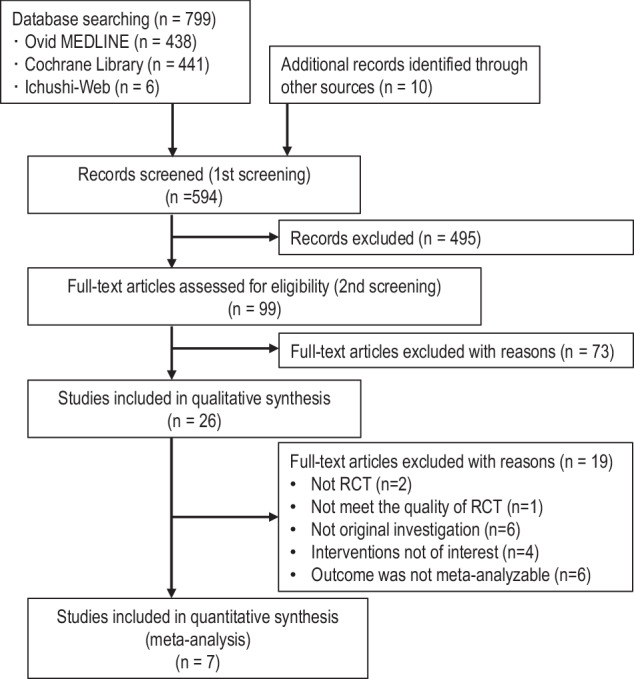
Flow diagram of study selection in this meta-analysis

Study-selection process was conducted in two stages. First, two independent reviewers screened titles and abstracts to identify potentially eligible studies. Second, the full texts of these studies were reviewed for inclusion. Discrepancies were resolved through discussion or consultation with a third reviewer. A PRISMA flow diagram summarizing the selection process is shown in Fig. 1.

### Data extraction

Data were independently extracted by YN using a standardized spreadsheet. Extracted variables included study title, year of publication, study design, population characteristics, intervention and control BP targets, baseline and achieved BP levels, number of participants, follow-up duration, and primary and secondary outcomes. Discrepancies were resolved by consensus or adjudicated by YNS. In the RESPECT trial, outcome data specific to patients aged ≥75 years were not reported in the original publication. Therefore, we directly contacted the corresponding author, who provided the relevant primary outcome data for this age subgroup [15]. Although the BPROAD trial [21] was published after the original systematic review was completed, we included it in a separate analysis to supplement the main findings.

### Risk of bias assessment

The quality of included RCTs was assessed using the Cochrane Risk of Bias 2.0 tool (ROB 2) [22], following the MINDS guidelines (2020 ver. 3.0) issued by the Japan Council for Quality Health Care. Two reviewers independently evaluated the risk of bias, and disagreements were resolved by discussion or a third reviewer.

### Statistical analysis

Statistical analyses were performed using Review Manager (version 5.4; the Cochrane Collaboration, Oxford, UK). A random-effect meta-analysis was performed using inverse-variance weighting to estimate pooled risk ratios (RRs) and 95% confidence intervals (CIs) for each outcome. Heterogeneity was quantified using I² statistic, with values of 0% indicating no heterogeneity, 25% indicating low heterogeneity, 25–50% indicating moderate heterogeneity, and 50% indicating high heterogeneity. P values of <0.05 were considered significant. Analyses were conducted for the primary population aged ≥75 years, and results from trials, including patients aged ≥70 years, were synthesized in a secondary analysis. Publication bias was assessed using funnel plots.

## Results

### Study selection

A total of 799 records were identified by searching the following databases: Ovid MEDLINE (*n* = 438), Cochrane Library (*n* = 441), and Ichushi-Web (*n* = 6). After removing duplicates, along with four RCTs [15, 18–20]) from manual searching and six RCTs [9–14] included in the previous systematic review [8], 594 records remained for title and abstract screening. After the initial screening step, 99 full-text articles were reviewed for eligibility.

Twenty-six articles were assessed using full-text screening. After applying the inclusion/exclusion criteria, 19 articles were excluded and reasons for full-text exclusion included non-RCT design (*n* = 2), substandard study quality (*n* = 1), secondary or non-original articles (n = 6), interventions not meeting the Population, Intervention, Comparison, and Outcome (PICO) criteria (*n* = 4), and outcomes not amenable to meta-analysis (*n* = 6). Ultimately, seven RCTs [9, 10, 15, 16, 19, 20, 23] were included in the final quantitative synthesis (Fig. 1).

### Study characteristics and quality assessment

Baseline characteristics of older participants in the seven RCTs [9, 10, 15, 16, 19, 20, 23] included in this meta-analysis are summarized in Table 1. Although all trials enrolled older adults with hypertensive having an elevated cardiovascular risk, the specific inclusion and exclusion criteria differed across the studies, shaping the risk profiles of their populations. All the studies targeted older participants, with four trials (the SPS3 [9], SPRINT [10], INFINITY [16], and RESPECT [15] trials) enrolling patients aged ≥75 years, and three trials (the PODCAST [19], STEP [23], and ESPRIT [20] trials) enrolling participants aged ≥70 years. Across all the trials, the participants were considered to be at high cardiovascular and cerebrovascular risks, but certain trials included populations with especially notable clinical backgrounds: the SPS3 [9] trial exclusively enrolled patients with a history of lacunar stroke. The PODCAST [19] enrolled patients with recent stroke. The INFINITY trial [16] enrolled patients with white matter lesions detected using brain MRI. The RESPECT trial [15] enrolled patients with ischemic stroke occurring within the preceding 3 years. In contrast, some trials deliberately excluded patients with certain comorbidities, whereas the SPRINT trial [10] excluded patients with type 2 diabetes mellitus and prior stroke. The INFINITY [16] and STEP [23] trials both excluded participants with a history of stroke. Other differences included baseline SBP (typically 141–148 mmHg), antihypertensive treatment targets (ranging from an SBP of <120 mmHg in the SPRINT [10] and ESPRIT trials [20], <125 mmHg in the PODCAST trial [19], and <130 mmHg in the SPS3 [9], INFINITY [16], RESPECT [15], and STEP [23] trials), and achieved BP levels, which were generally 10–20 mmHg lower in the intensive treatment groups. Comorbidities, such as chronic kidney disease, coronary artery disease, and cognitive dysfunction, have been reported. Gait speed and physical function were evaluated using the SPRINT [10] and INFINITY trials [16]. Cognitive function was assessed using the PODCAST [19] and INFINITY [16] trials. These heterogeneities in trial design and population characteristics underscore the importance of a pooled analysis to clarify the benefits and safety of intensive BP lowering in older hypertensive individuals, especially those aged ≥75 years. The risk of bias in the included studies is summarized in Supplementary Table 2. Using the RoB 2 tool [22], the overall risk of bias was evaluated as low in four trials and some concerns in three trials.

**Table 1:** Baseline characteristics of older participants in studies included in the meta-analysis

Study	Publication	Age	Target BP				Patients		Patients age		Follow-up	Inclusion criteria		Study-specific exclusion criteria		% of HT	% of DM	% of stroke	% of CAD	Mean Cr mg/dL, eGFR or % of CKD	Frailty-related outcomes other than the cardiovasucular outcome	Mesurement of BP	Baseline BP		Achieved BP			
			More intensive		Less intensive		More intensive	Less intensive	More intensive	Less intensive		Disease	BP	Disease	Renal function								SBP	DBP	More intensive		Less intensive	
			SBP	DBP	SBP	DBP																			SBP	DBP	SBP	DBP
	Year	(Years)	(mm Hg)				(*n*)		(Years)		(Years)												(mm Hg)		(mm Hg)			
SPS3 [9]	2015	≥75^a^	<130	–	130–149	–	248	246	79.9		3.7	Lacunar stroke within the previous 180 days	SBP≥130 or those taking antihypertensive medication	–	eGFR <40	87.7^b^	26.9	14.2 vs 15.0	10.3 vs 10.5	eGFR 66.0	N/A	OBP	144.4	N/A	125.2	N/A	137.1	N/A
SPRINT [10]	2016	≥75^a^	<120	–	<140	–	1317	1319	79.8	79.9	3.14	HT	SBP≥130	DM, Stroke, Orthostatic hypotension	eGFR <20	>90^b^	0	0	25.7 vs 23.4	Cr 1.1 vs 1.1 eGFR 63.4 vs 63.3 % of CKD 44.3 vs 44.7	gait speed 0.90 vs 0.92 m/s % of <0.80 m/s: 28.2 vs 28.0% % of Frail (frailty index >0.21): 33.4 vs 28.4%	AOBP	141.6 vs 141.6 (AOBP)	71.5 vs 70.9 (AOBP)	123.4 (AOBP)	62.0 (AOBP)	134.8 (AOBP)	67.2 (AOBP)
PODCAST [19]	2017	>70	<125	–	<140	–	41	42	73	75.1	2.0	HT, previous stroke	SBP 125–170	–	eGFR <45	84.6%	29.3 vs 11.9	7.3 vs 11.9	26.8 vs 21.4	N/A	Cognitive function (ACE-R): 80.8 vs 84.4	OBP	147.1 (145.9 vs 148.3)	82.1 (82.5 vs 81.7)	130.0	72.9	140.5	77.4
INFINITY [16]	2019	≥75	≤130	–	≤145	–	99	100	80.9	80.3	3	HT with visible white matter hyperintensity lesions on screening magnetic brain imaging	SBP 150–170 mmHg (if taking >2 antihypertensive drugs) or 170 mmHg < on 0 to 1 antihypertensive drug at screening	uncontrolled DM, Stroke	eGFR <25	100	14.1 vs 18^c^	0	40.2 vs 39	Cr 1.09 22.2 vs 24.2	gait speed: 0.98 vs 0.99 m/s Supine-to-sit: 3.0 vs 2.8 s FTSST: 10.7 vs 10.2 s Unipedal balance: 12.2 vs 12.9 s MMSE 28.1 vs 28.3	24-h ABPM OBP	149.7 vs 152.0 (OBP) 147.8 vs 150.3 (24-h ABPM)	73.9 vs 77.3 (OBP) 73.2 vs 74.9 (24-h ABPM)	127.7 (24-h ABPM)	N/A	144.0 (24-h ABPM)	N/A
RESPECT [15]	2019	≥75	<130	<80	<140	<90	147	158	78.8	78.5	3.74	HT, stroke within the previous 3 years	SBP 130–179 or DBP 80–109	Stroke within previous month	Cr >2.0	100	19.7 vs 27.2	100	4.8 vs 3.8	Cr 0.86 vs 0.83 % of CKD 8.2 vs 7.6	N/A	OBP	146.5 (146.1 vs 146.9)	79.8 (80.2 vs 79.4)	126.5	73.5	133.4	69.4
STEP [23]	2021	≥70	110 to <130	–	130 to <150	–	1023	1032	N/A	N/A	3.34	HT	SBP 140–190 mm, or those taking antihypertensive medication	Stroke (not including lacunar infarction and transient ischemic attack)	eGFR <30 Cr >2.5	100	18.9 vs 19.4^d^	N/A	6.3 vs 6.4^d^	% of CKD 2.4 vs 2.3^d^	N/A	OBP	146.1 vs 146.0^d^	82.7 vs 82.3^d^	126.7^d^	76.4^d^	135.9^d^	79.2^d^
ESPRIT [20]	2024	≥70	<120	–	<140	–	1365	1384	N/A	N/A	3.4	HT, Prior cardiovascular disease or at least two cardiovascular risk factors	SBP 130–180	–	eGFR <45	100	38.8 vs 38.7^d^	27.0 vs 26.7^d^	29.0 vs 28.8^d^	eGFR 83.2 vs 83.5^d^ % of CKD 6.0 vs 6.0^d^	N/A	OBP	146.8 vs 147.0^d^	82.8 vs 82.9^d^	119.1^d^	N/A	134.8^d^	N/A

*ABPM* ambulatory blood pressure monitoring, *CAD* coronary artery disease, *AOBP* automated office blood pressure, *CKD* chronic kidney disease, *Cr* creatinine, *DM* diabetes mellitus, *eGFR* estimated glomerular filtration rate, *HT* hypertension, *N/A* not available, *OBP* office blood pressure, *SBP* systolic blood pressure, *DBP* diastolic blood pressure, *MMSE* Mini Mental State Examination

^a^Subgroup analysis or post hoc analysis of the original trial

^b^Those taking antihypertensive medications

^c^Those taking antihyperglycemic medications

^d^Date from patients of all ages

### Effects of intensive BP-lowering treatments on all-cause mortality and cardiovascular events

**Fig. 2:**
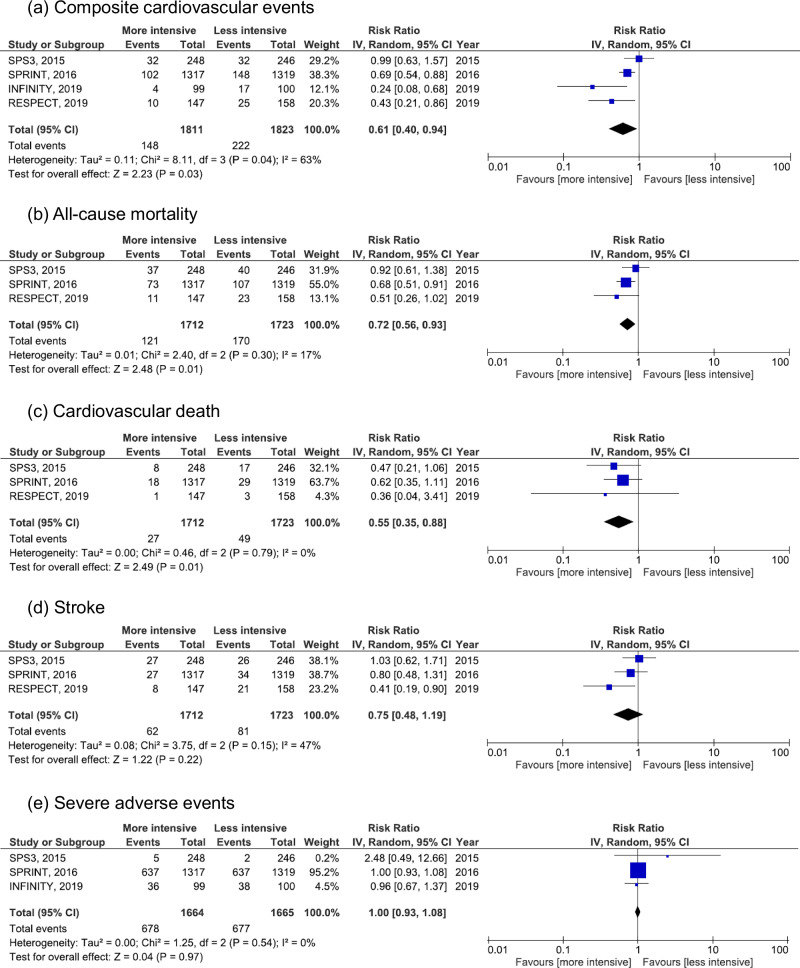
Effect of intensive BP lowering on risk of cardiovascular events and severe adverse effects in patients with hypertension aged ≥ 75 years. **a** Composite cardiovascular events, **b** all-cause mortality, **c** cardiovascular death, **d** stroke, and **e** severe adverse events. Boxes and horizontal lines represent risk ratio (RR) and 95% confidence interval (CI) for each trial. The size of boxes is proportional to the weight of that trial result. Diamonds represent 95% CI for pooled estimates of effect and are centered on pooled RR

A meta-analysis of four RCTs evaluating intensive versus standard BP lowering in patients with hypertension aged ≥75 years demonstrated significant clinical benefits of intensive BP targets. Intensive BP control significantly reduced the risk of composite cardiovascular events (RR: 0.61; 95% CI: 0.40–0.94; *P* = 0.03), although statistical heterogeneity was substantial (I² = 63%, *P* = 0.04). All-cause mortality was also significantly lower in the intensive treatment group (RR: 0.72; 95% CI: 0.56–0.93; *P* = 0.01) with low heterogeneity (I² = 17%, *P* = 0.30). Similarly, cardiovascular mortality was significantly reduced (RR: 0.55; 95% CI: 0.35–0.88; *P* = 0.01), with no heterogeneity observed (I² = 0%, *P* = 0.79). In contrast, the incidence of stroke did not differ significantly between groups (RR: 0.75; 95% CI: 0.48–1.19; *P* = 0.22), with moderate heterogeneity (I² = 47%, *P* = 0.15). Importantly, there was no significant increase in serious adverse events associated with intensive BP lowering (RR: 1.00; 95% CI: 0.93–1.08; *P* = 0.97), and no heterogeneity was observed (I² = 0%, *P* = 0.54). The results are summarized in Fig. 2, and potential publication bias was assessed using funnel plots presented in Supplementary Fig. 1.

The BPROAD trial [21] was published after completion of the initial analysis. A subgroup analysis of patients aged ≥80 years was included in the meta-analysis. The results remained consistent, showing that intensive BP lowering continued to significantly reduce the risk of composite cardiovascular events (RR: 0.63; 95% CI: 0.44–0.90; *P* = 0.01; I² = 51%, *P* = 0.09), as shown in Supplementary Fig. 2. In contrast, a sensitivity analysis restricted to stroke survivors—based on three trials (the SPS3 [9], PODCAST [19], and RESPECT [15] trials)—did not show a statistically significant benefit for intensive BP control on composite cardiovascular outcomes (RR: 0.62; 95% CI: 0.30–1.30; *P* = 0.21; I² = 59%, *P* = 0.09), as presented in Supplementary Fig. 3.

In a secondary analysis that included trials enrolling participants aged ≥70 years, the benefits of intensive BP lowering were generally consistent with those observed in the ≥75-year population. Intensive treatment significantly reduced the risk of composite cardiovascular events (RR: 0.72; 95% CI: 0.57–0.90; *P* = 0.003), all-cause mortality (RR: 0.74; 95% CI: 0.58–0.93; *P* = 0.009), and cardiovascular mortality (RR: 0.56; 95% CI: 0.36–0.89; *P* = 0.01). No significant reduction was observed for stroke incidence (RR: 0.76; 95% CI: 0.55–1.04; *P* = 0.09), and there was no increase in serious adverse events (RR: 1.00; 95% CI: 0.92–1.08; *P* = 0.93) (Supplementary Fig. 4).

## Discussion

This systematic review and meta-analysis demonstrate that, in patients with hypertension aged ≥75 years, intensive SBP lowering to a target of <130 mmHg is associated with significant reductions in composite cardiovascular events, all-cause mortality, and cardiovascular mortality, without increasing the risk of serious adverse events. These findings support the validity of a more aggressive BP target in this population, and provide updated evidence to inform future clinical guidelines.

Compared with the previous systematic review conducted in 2019 by Takami et al. [8], which supported an SBP target of <140 mmHg based on six RCTs, our current analysis includes newer, larger-scale studies, such as the STEP [23] and ESPRIT [20] trials, which adopted lower BP targets. Importantly, all the seven trials defined the intensive treatment arm as targeting an SBP of <130 mmHg, enabling a focused assessment of this more stringent goal. Furthermore, we restricted our primary analysis to trials that enrolled patients aged ≥75 years, offering more specific insights into the very old population.

In a 2019 review [8], intensive BP control was associated with significant reductions in all-cause and cardiovascular mortality but not in major cardiovascular events. In contrast, the present analysis shows a statistically significant benefit in composite cardiovascular outcomes (RR: 0.61; 95% CI: 0.40–0.94), as well as mortality endpoints. These differences may be attributed to the inclusion of newer trials with stricter BP targets, longer follow-up durations, and higher statistical power. Moreover, our findings align with the BPLTTC individual-level meta-analysis [24], which demonstrated that lowering BP reduces the risk of major cardiovascular events in individuals aged <85 years, with no evidence of harm. Although the reduction was not statistically significant in those aged ≥85 years, the trend remained consistent (RR: 0.99; 95% CI: 0.87–1.12; *P* = 0.05).

Importantly, the benefits observed in our analysis were not offset by an increased incidence of serious adverse events, suggesting that intensive BP control is generally safe in well-selected older adults. This counters a commonly held concern in geriatric care that tighter BP control may increase the risk of falls, syncope, or renal impairment. However, it must be emphasized that the trials included in this review generally excluded patients with advanced frailty, dementia, or institutionalization, thereby limiting the generalizability of the findings to such high-risk subpopulations.

A notable contribution of this study is the inclusion of the recently published BPROAD trial [21] in a sensitivity analysis. When subgroup data from patients aged ≥80 years in this trial were incorporated, the protective effect of intensive BP lowering on composite cardiovascular outcomes remained robust (RR: 0.63; 95% CI: 0.44–0.90). Conversely, in separate analyses restricted to patients with a history of stroke (the SPS3 [9], PODCAST [19], and RESPECT [15] trials), the benefit of intensive BP control was not statistically significant (RR: 0.64; 95% CI: 0.30–1.38). These results suggest that although intensive BP-lowering is effective in the general older hypertensive population, its efficacy in patients with post-stroke requires further validation.

Our analysis also included a supplementary meta-analysis of patients aged ≥70 years, yielding results consistent with those from the ≥75-year cohort. Intensive BP control reduces the risk of cardiovascular events and mortality, without increasing the incidence of adverse events. This strengthens harmonizing BP targets across age groups and supports recent trends in international guidelines, such as the 2024 European Society of Cardiology guidelines [25], which no longer proposes distinct BP targets for most older adults up to 85 years of age. The average age of participants in the included RCTs generally ranged from 75 to 80 years, and the proportion of patients aged ≥85 years was likely small. When considered alongside the findings of the BPLTTC meta-analysis [24], which showed no statistically significant benefit of BP lowering in the subgroup aged ≥85 years, this underscores the limited evidence base for intensive BP treatment in the oldest-old population. Therefore, we emphasize that caution is warranted when applying these findings to individuals with markedly advanced age or limited life expectancy.

Nonetheless, significant clinical heterogeneity was observed across the included studies, particularly in terms of baseline patient characteristics. The SPS3 [9] and PODCAST [19] trials focused on stroke survivors, the INFINITY trial [16] included patients with white matter hyperintensities on brain MRI, and the RESPECT trial [15] targeted individuals within 3 years of ischemic stroke. In contrast, the SPRINT [10] trial excluded patients with diabetes and prior stroke, whereas the STEP [23] and INFINITY [16] trials excluded patients with a history of stroke. Other factors, such as the prevalence of coronary artery disease, diabetes, and chronic kidney disease, varied widely, and the eGFR cut-offs for exclusion ranged from <20 to <45 mL/min/1.73 m². These variations should be considered when extrapolating our findings to diverse patient populations.

Physiologically, older adults often exhibit increased vascular stiffness, altered autonomic regulation, and impaired renal function, which may modify their hemodynamic responses to antihypertensive therapy. Despite these concerns, our results support the clinical value of achieving an SBP of <130 mmHg in older patients, particularly given their high absolute risk of cardiovascular events. Although relative risk reduction with antihypertensive therapy may diminish with age, absolute risk reduction often becomes more pronounced in older age groups [24].

One of the critical clinical challenges in the management of hypertension is clinical inertia, which is the failure to initiate or intensify treatment when indicated. Ambiguity surrounding age-based BP targets has contributed to therapeutic hesitation and inconsistent implementation of guideline-recommended therapies [26]. Our findings suggest that a simplified approach using a common SBP target in older and younger patients may enhance treatment clarity and promote consistent care delivery.

This study has several strengths. First, it provides focused synthesis of high-quality evidence derived from RCTs specifically enrolling individuals aged ≥75 years, a population frequently under-represented in prior meta-analyses, along with those aged ≥70 years. Second, we included only RCTs with a clearly defined SBP target of <130 mmHg in the intensive treatment group to ensure a rigorous comparison. Third, the sensitivity analyses incorporating the BPROAD trial [21] and stroke-specific cohorts offer additional granularity and relevance in clinical practice.

On the other hand, this study has several important limitations. First, regarding comorbidity-specific recommendations, the current evidence base is limited. Owing to limited data availability, we did not perform an analysis stratifying the patients based on the presence or absence of diabetes, chronic kidney disease, and prior cardiovascular disease. Second, most of the included trials excluded patients with severe frailty, dementia, institutionalization, or end-of-life conditions, thereby limiting generalizability to the most vulnerable populations. The SPRINT trial conducted post-hoc frailty stratification [10] and reported no attenuation of cardiovascular benefits in frail patients; however, this remains an area for further studies. Third, given that the proportion of patients aged ≥85 years enrolled in these RCTs was likely small, the generalizability of our findings to this age group is limited. Fourth, the heterogeneity in outcome definitions, treatment protocols, and comorbidity profiles complicates direct comparisons and underscores the need for individualized clinical judgment. Although some studies [16, 19, 27, 28] have incorporated assessments of physical or cognitive function, heterogeneity in outcome measures precluded meta-analyses for these domains. Notably, there is currently no conclusive evidence that intensive BP-lowering directly contributes to the preservation of physical or cognitive function in older patients. For example, the SPRINT-MIND sub-study [27] showed a lower incidence of mild cognitive impairment with intensive BP control, but no significant difference in the incidence of probable dementia. Similarly, the INFINITY trial [16], which targeted older patients with white matter lesions, did not demonstrate a significant improvement in cognitive outcomes despite reductions in ambulatory BP. These findings underscore the complexity of the relationship between BP control and brain health in older adults. Dedicated RCTs with cognitive function or physical performance as primary endpoints are urgently needed to clarify the potential non-cardiovascular benefits and risks of intensive BP-lowering in this population. Fifth, although the target SBP differed slightly among the studies, the achieved SBP levels in the intensive treatment arms consistently fell between 120 and 130 mmHg. The current meta-analysis supports the cardiovascular benefits of this moderate level of BP lowering adults with hypertension aged ≥75 years. However, the safety and efficacy of more aggressive BP targets—such as SBP < 110 mmHg—remain unclear, as no major RCTs have directly examined such thresholds in older adults. Indeed, the potential for harm associated with excessive BP reduction in the elderly has been raised in prior studies. For example, the J-curve phenomenon has been documented, particularly in older adults at high risk of cerebral or coronary artery disease, in whom intensive BP lowering (SBP < 120 mmHg) may paradoxically increase the risk of cardiovascular events [29]. Furthermore, observational studies in nursing home residents have reported adverse outcomes associated with SBP levels <110 mmHg [30, 31]. A large-scale cohort study in China also suggested that among individuals aged ≥85 years with frailty, SBP < 120 mmHg was associated with an increased risk of mortality. Most international guidelines do not define a strict lower boundary for SBP targets in older adults. However, based on the available evidence, we consider a lower limit of SBP target around 120 mmHg to be a reasonable therapeutic goal, while avoiding overtreatment that may lead to harm in vulnerable subgroups. Finally, this review focused on the magnitude of BP lowering but did not address the quality of BP control, particularly BP variability. Increased arterial stiffness, impaired baroreflex sensitivity, and a predisposition to dehydration in older adults can lead to greater BP fluctuations, which may, in turn, increase the risk of adverse events such as orthostatic hypotension, syncope, and falls [3]. Future research should explore not only BP targets but also the quality of BP control, including variability, to better guide individualized treatment strategies in this population.

In conclusion, this updated meta-analysis provides strong evidence supporting the safety and efficacy of intensive BP control (SBP, <130 mmHg) in patients with hypertension aged ≥75 years. Our findings argue against the use of higher BP targets in the absence of compelling contraindications, and support a more unified approach to BP management across age groups. Future guidelines should consider retiring arbitrary age thresholds in favor of individualized, risk-based decision making that acknowledges the growing heterogeneity of the older adult population. Further studies are warranted to define optimal BP targets in frail, institutionalized, or cognitively impaired individuals, who remain underrepresented in the existing evidence.

## Supplementary information

Supplementary Material
