# Supplementary material for: Targeting a systolic blood pressure of <130 mmHg is beneficial in adults with hypertension aged ≥75 years: a systematic review and meta-analysis
Source: Hypertens Res. 2025 Aug 12;48(10):2527–36. doi: 10.1038/s41440-025-02302-z (PMC12497641; doi:10.1038/s41440-025-02302-z)
Supplement: Supplementary file 1 — Supplementary Material [file 41440_2025_2302_MOESM1_ESM.docx]

**SUPPLEMENTARY MATERIAL**

**Targeting a Systolic Blood Pressure of <130 mmHg is Beneficial in Adults with Hypertension Aged ≥75 Years: A Systematic Review and Meta–Analysis**

Yoichi Nozato^1)2)^, Yume Nohara-Shitama^3)^, Takuro Kubozono^4)^, Hiroshi Akasaka^1)5)^, Yoichi Takami^1)^, Hisatomi Arima^6)^, Atsushi Sakima^7)^, Koichi Yamamoto^1)^

1) Department of Geriatric and General Medicine, The University of Osaka, Graduate School of Medicine, Osaka, Japan

2) Toyonaka Municipal Hospital, Department of Geriatric and General Medicine, Osaka, Japan

3) Division of Cardiovascular Medicine, Department of Internal Medicine, Kurume University School of Medicine, Fukuoka, Japan

4) Department of Cardiovascular Medicine and Hypertension, Graduate School of Medical and Dental Sciences, Kagoshima University, Kagoshima, Japan

5) Department of Hygiene and Preventive Medicine, Iwate Medical University School of Medicine, Iwate, Japan

6) Department of Preventive Medicine and Public Health, Faculty of Medicine, Fukuoka University, Fukuoka, Japan

7) Health Administration Center, University of the Ryukyus, Okinawa, Japan

**Supplementary Table 1.**

Search formula in Ovid MEDLINE

Date of search: May 30 2024

| # | Search formula | Results |
| --- | --- | --- |
| 1 | hypertension/ | 263681 |
| 2 | blood pressure/ | 295136 |
| 3 | or/1-2 | 480515 |
| 4 | antihypertensive agents/ or (antihypertensive or anti-hypertensive).ti,ab. | 104431 |
| 5 | adrenergic alpha-antagonists/ or adrenergic alpha-1 receptor antagonists/ or adrenergic beta-antagonists/ or adrenergic beta-1 receptor antagonists/ or (adrenergic alpha-antagonist* or adrenergic alpha-antagonist*).ti,ab. | 56684 |
| 6 | angiotensin converting enzyme inhibitors/ or angiotensin converting enzyme inhibitor*.ti,ab. | 46735 |
| 7 | angiotensin receptor antagonists/ or angiotensin ii type 1 receptor blockers/ or angiotensin receptor antagonist*.ti,ab. | 21076 |
| 8 | calcium channel blockers/ or calcium channel blocker*.ti,ab. | 47213 |
| 9 | diuretics/ or diuretic*.ti,ab. | 58293 |
| 10 | Aldosterone/ | 25703 |
| 11 | Mineralocorticoids/ | 2201 |
| 12 | 4 or 5 or 6 or 7 or 8 or 9 or 10 or 11 | 296224 |
| 13 | exp Cardiovascular Diseases/ | 2790533 |
| 14 | exp Heart Failure/ | 153967 |
| 15 | exp Kidney Diseases/ or exp Kidney Failure, Chronic/ | 587987 |
| 16 | stroke/ or brain infarction/ or cerebral infarction/ or stroke, lacunar/ | 166093 |
| 17 | (death* or mortalit* or transient ischemic attack* or TIA or cerebrovascular or (heart adj (disease* or failure*)) or ((renal or nephro* or kidney) adj2 (disease* or failure* or disorder*))).ti,ab. | 2426320 |
| 18 | 13 or 14 or 15 or 16 or 17 | 4866314 |
| 19 | (intensive or strict or tight or low or optimal or active).ti,ab. | 4966173 |
| 20 | 3 and 12 and 18 and 19 | 13836 |
| 21 | limit 20 to (yr="2017 -Current" and english and randomized controlled trial) | 438 |

Search formula in Ovid MEDLINE

Date of search: May 30 2024

| # | Search formula | Results |
| --- | --- | --- |
| 1 | MeSH descriptor: [Hypertension] explode all trees | 25318 |
| 2 | (hypertension):ti,ab,kw | 74997 |
| 3 | #1 OR #2 | 74999 |
| 4 | MeSH descriptor: [Antihypertensive Agents] explode all trees | 10838 |
| 5 | (antihypertensive or anti-hypertensive):ti,ab,kw | 22917 |
| 6 | MeSH descriptor: [Adrenergic alpha-Antagonists] explode all trees | 1559 |
| 7 | MeSH descriptor: [Adrenergic alpha-1 Receptor Antagonists] explode all trees | 370 |
| 8 | MeSH descriptor: [Adrenergic beta-Antagonists] explode all trees | 5812 |
| 9 | MeSH descriptor: [Adrenergic beta-1 Receptor Antagonists] explode all trees | 277 |
| 10 | ("adrenergic alpha-antagonist" or "adrenergic alpha-antagonists" or "adrenergic alphaantagonist" or "adrenergic alphaantagonists"):ti,ab,kw | 1194 |
| 11 | MeSH descriptor: [Angiotensin-Converting Enzyme Inhibitors] explode all trees | 5304 |
| 12 | ("angiotensin converting enzyme inhibitor" or "angiotensin converting enzyme inhibitors"):ti,ab,kw | 7693 |
| 13 | MeSH descriptor: [Calcium Channel Blockers] explode all trees | 3527 |
| 14 | ("calcium channel blocker" or "calcium channel blockers"):ti,ab,kw | 5505 |
| 15 | MeSH descriptor: [Angiotensin Receptor Antagonists] explode all trees | 3033 |
| 16 | MeSH descriptor: [Angiotensin II Type 1 Receptor Blockers] explode all trees | 1660 |
| 17 | ("angiotensin receptor antagonist" or "angiotensin receptor antagonists" or "angiotensin II type 1 receptor blocker" or "angiotensin II type 1 receptor blockers"):ti,ab,kw | 3825 |
| 18 | MeSH descriptor: [Diuretics] explode all trees | 4118 |
| 19 | (diuretic*):ti,ab,kw | 10444 |
| 20 | MeSH descriptor: [Mineralocorticoid Receptor Antagonists] explode all trees | 929 |
| 21 | ("mineralocorticoid receptor antagonist" or "mineralocorticoid receptor antagonists" or "mineralocorticoid receptor blocker" or "mineralocorticoid receptor blockers" or "aldosterone receptor antagonist" or "aldosterone receptor antagonists" or "aldosterone receptor blocker" or "aldosterone receptor blockers"):ti,ab,kw | 1405 |
| 22 | #4 or #5 or #6 or #7 or #8 or #9 or #10 or #11 or #12 or #13 or #14 or #15 or #16 or #17 or #18 or #19 or #20 or #21 | 45045 |
| 23 | MeSH descriptor: [Cardiovascular Diseases] explode all trees | 155891 |
| 24 | MeSH descriptor: [Heart Failure] explode all trees | 14525 |
| 25 | MeSH descriptor: [Kidney Diseases] explode all trees | 23476 |
| 26 | MeSH descriptor: [Kidney Failure, Chronic] explode all trees | 6065 |
| 27 | MeSH descriptor: [Stroke] explode all trees | 17453 |
| 28 | MeSH descriptor: [Brain Infarction] explode all trees | 1886 |
| 29 | MeSH descriptor: [Cerebral Infarction] explode all trees | 1667 |
| 30 | MeSH descriptor: [Stroke, Lacunar] explode all trees | 69 |
| 31 | ((death or mortalit* or cerebrovascular or cardiovascular)):ti,ab,kw | 254588 |
| 32 | #23 or #24 or #25 or #26 or #27 or #28 or #29 or #30 or #31 | 361460 |
| 33 | ((elder* or aged or old or older or oldest or senior* or geriatric*)):ti,ab,kw | 759094 |
| 34 | MeSH descriptor: [Aged] explode all trees | 275455 |
| 35 | MeSH descriptor: [Aged, 80 and over] explode all trees | 70882 |
| 36 | MeSH descriptor: [Frail Elderly] explode all trees | 1134 |
| 37 | #34 or #35 or #36 | 275455 |
| 38 | ((intensive or strict or tight or low or optimal or active)):ti,ab,kw | 445053 |
| 39 | MeSH descriptor: [Randomized Controlled Trials as Topic] explode all trees | 54940 |
| 40 | (randomiz* or randomis* or randomly):ti,ab,kw | 1312517 |
| 41 | #39 or #40 | 1312633 |
| 42 | #3 and #22 and #32 and #33 and #37 and #38 and #41 with Publication Year from 2017 to 2024, in Trials | 391 |

Search formula in Ichushi Web

Date of search: May 30 2024

| # | Search formula | Results |
| --- | --- | --- |
| 1 | 高血圧/TH | 132315 |
| 2 | 高齢者/TH | 92480 |
| 3 | 降圧剤/TH | 88435 |
| 4 | "Adrenergic Alpha-Antagonists"/TH | 14733 |
| 5 | "Adrenergic Beta-Antagonists"/TH | 24773 |
| 6 | "Calcium Channel Blockers"/TH | 45871 |
| 7 | "Angiotensin-Converting Enzyme Inhibitors"/TH | 20481 |
| 8 | "Angiotensin II Type 1 Receptor Blockers"/TH | 22924 |
| 9 | 利尿剤/TH | 25425 |
| 10 | "Mineralocorticoid Receptor Antagonists"/TH | 5322 |
| 11 | #3 or #4 or #5 or #6 or #7 or #8 or #9 or #10 | 149387 |
| 12 | 心臓血管疾患/TH | 1467911 |
| 13 | 心不全/TH | 76075 |
| 14 | 脳卒中/TH | 111761 |
| 15 | 頭蓋内出血/TH | 62599 |
| 16 | 腎機能障害/TH | 207418 |
| 17 | 死亡/TH | 58758 |
| 18 | #12 or #13 or #14 or #15 or #16 or #17 | 1676366 |
| 19 | #1 and #2 and #11 and #18 | 1042 |
| 20 | #19 and ランダム化比較試験/TH | 106 |
| 21 | DT=2017:2024 | 2610892 |
| 22 | #20 and #21 | 6 |

**Supplementary Table 2. Summary for risk of bias in studies included in this meta-analysis.**

| Study, year | Bias domain^＊^ | | | | |  |
| --- | --- | --- | --- | --- | --- | --- |
|  | Bias arising from randomization process | Bias due to deviations from intended interventions | Bias due to missing outcome data | Bias in measurement of outcome | Bias in selection of reported results | Overall bias |
| SPS3, 2015 | 0 | 0 | 0 | 0 | 0 | 0 |
| SPRINT, 2016 | 0 | 0 | 0 | 0 | 0 | 0 |
| PODCAST, 2017 | 0 | 0 | 0 | 0 | 0 | 0 |
| INFINITY, 2019 | -1 | 0 | 0 | 0 | 0 | -1 |
| RESPECT, 2019 | 0 | 0 | 0 | 0 | 0 | 0 |
| STEP, 2021 | 0 | 0 | -1 | 0 | 0 | -1 |
| ESPRIT, 2024 | 0 | 0 | -1 | 0 | 0 | -1 |

0: low risk, -1: some concerns

*We assessed the risk of bias for each individual randomized controlled trial included in the meta-analysis using version 2 of the Cochrane tool for assessing risk of bias in randomized trials (RoB 2).

**Supplementary Figure 1. Funnel plots of each meta-analysis for trials enrolling patients aged ≥75 years**


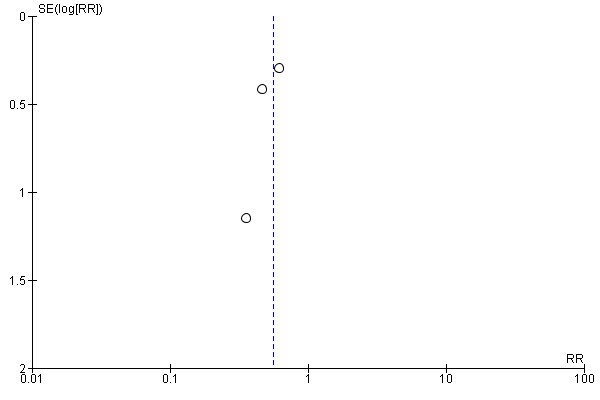

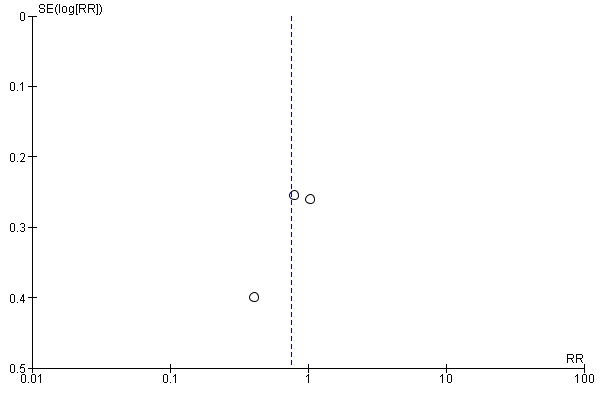

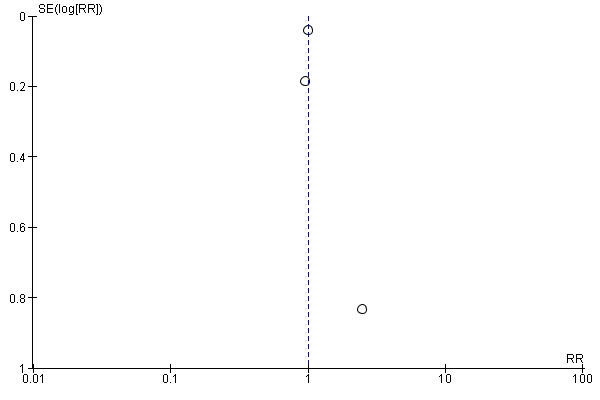

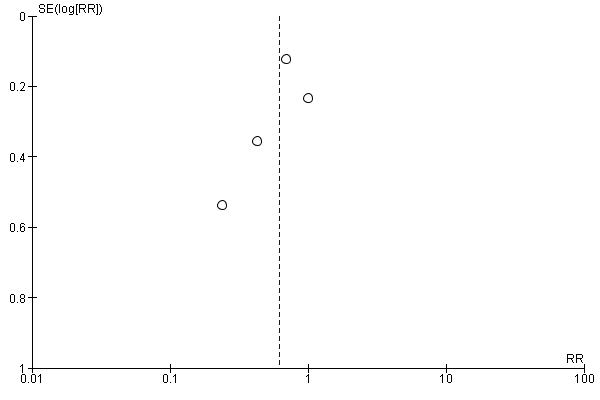

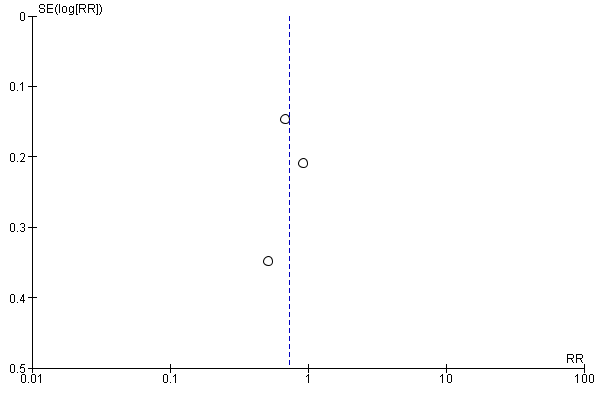


(a) Composite cardiovascular events

(b) All-cause mortality

(c) Cardiovascular death

(d) Stroke

(e) Severe adverse events

**Supplementary Figure 2. Effect of intensive BP lowering on risk of composite cardiovascular events in patients with hypertension aged ≥75 years including the BPROAD trial**


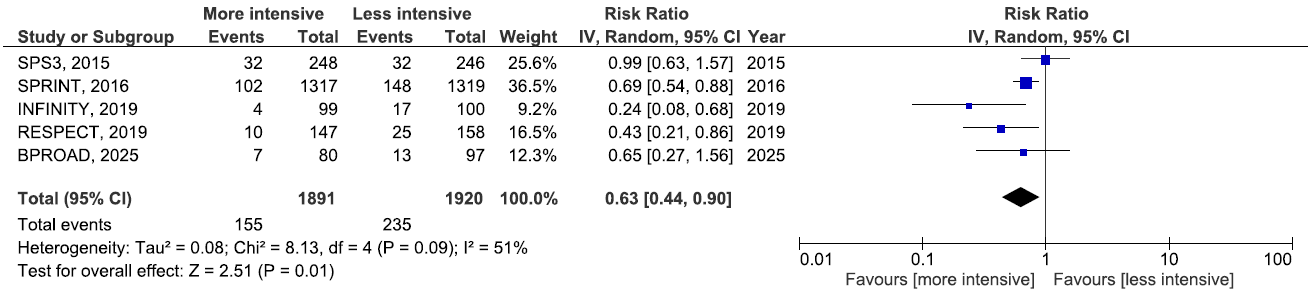


**Supplementary Figure 3. Effect of intensive BP lowering on the risk of composite cardiovascular events in patients with hypertension aged ≥75 years with a history of stroke**


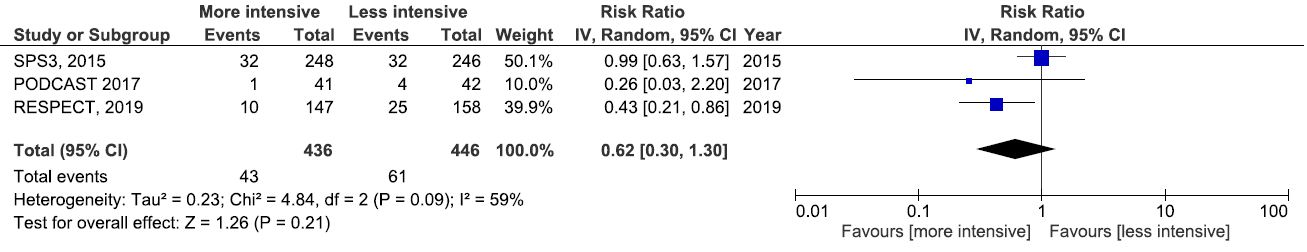


**Supplementary Figure 4. Effect of intensive BP lowering on the risk of composite cardiovascular events in patients with hypertension aged ≥70 years**


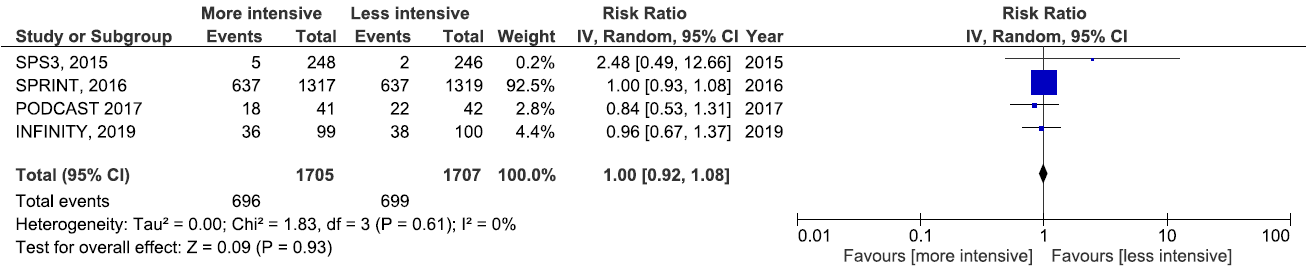

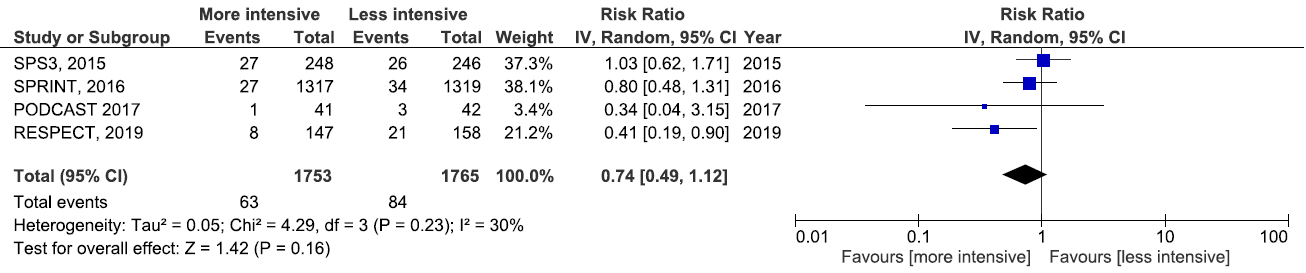

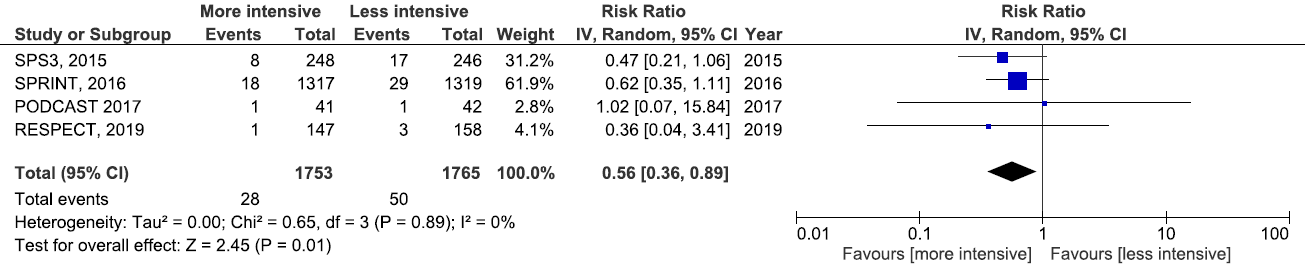

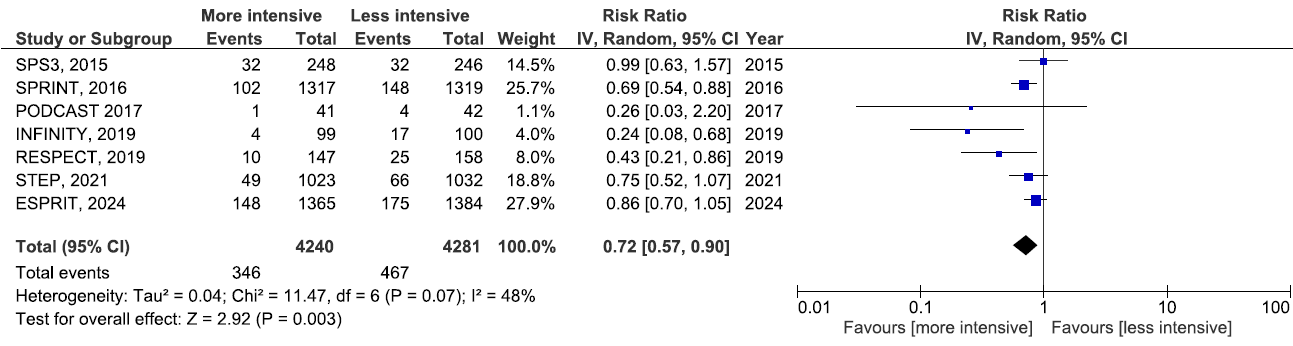

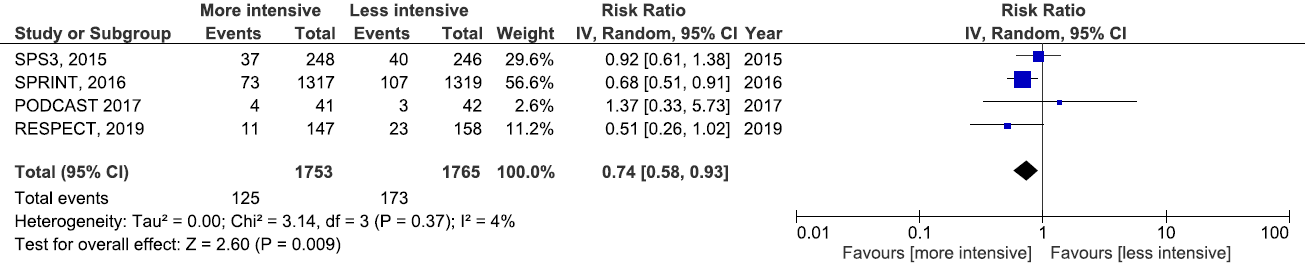


(e) Severe adverse events

(b) All-cause mortality

(c) Cardiovascular death

(d) Stroke

(a) Composite cardiovascular events
